# Photoinduced Polymerization of Eugenol-Derived Methacrylates

**DOI:** 10.3390/molecules25153444

**Published:** 2020-07-29

**Authors:** Samantha Molina-Gutiérrez, Sara Dalle Vacche, Alessandra Vitale, Vincent Ladmiral, Sylvain Caillol, Roberta Bongiovanni, Patrick Lacroix-Desmazes

**Affiliations:** 1 Institut Charles Gerhardt Montpellier (ICGM), University of Montpellier, CNRS, ENSCM, 34095 Montpellier, France; samantha.molina-gutierrez@enscm.fr (S.M.-G.); vincent.ladmiral@enscm.fr (V.L.); sylvain.caillol@enscm.fr (S.C.); 2 Department of Applied Science and Technology, Politecnico di Torino, Corso Duca degli Abruzzi 24, 10129 Torino, Italy; sara.dallevacche@polito.it (S.D.V.); alessandra.vitale@polito.it (A.V.)

**Keywords:** biobased monomer, photoinduced-polymerization, eugenol-derived methacrylate

## Abstract

Biobased monomers have been used to replace their petroleum counterparts in the synthesis of polymers that are aimed at different applications. However, environmentally friendly polymerization processes are also essential to guarantee greener materials. Thus, photoinduced polymerization, which is low-energy consuming and solvent-free, rises as a suitable option. In this work, eugenol-, isoeugenol-, and dihydroeugenol-derived methacrylates are employed in radical photopolymerization to produce biobased polymers. The polymerization is monitored in the absence and presence of a photoinitiator and under air or protected from air, using Real-Time Fourier Transform Infrared Spectroscopy. The polymerization rate of the methacrylate double bonds was affected by the presence and reactivity of the allyl and propenyl groups in the eugenol- and isoeugenol-derived methacrylates, respectively. These groups are involved in radical addition, degradative chain transfer, and termination reactions, yielding crosslinked polymers. The materials, in the form of films, are characterized by differential scanning calorimetry, thermogravimetric, and contact angle analyses.

## 1. Introduction

The need for more environmentally friendly materials and processes has led to the development of suitable biobased building blocks to produce polymers [1]. However, the use of energy-efficient polymerization techniques is also paramount. Photoinduced polymerization is a suitable option, as it allows fast processes, low energy consumption, room temperature reactions, and solvent-free conditions with the concomitant reduction or elimination of volatile organic compounds (VOCs) [2]. Thanks to these advantages, it has found wide application in industrial processes. It is an established technique in the fields of coatings, inks, adhesives, and wood finishing [3]. Products from photopolymerization are present in everyday life, such as contact lenses [4], filling for dental cavities [5], and credit cards [6].

Photopolymerization processes are also characterized by spatial and temporal control, which means that they only occur in the irradiated area and they are stop-and-go reactions, i.e., they start and stop simply by switching on and off the light [7]. Therefore, they are key reactions for the emerging additive manufacturing technologies [8,9,10]. For most applications, conventional radical reactions (proceeding via propagation of macromolecular radicals after initiation triggered by irradiation) are employed. Most monomers do not produce any radical initiating species with sufficiently high yields under light irradiation; thus, a photoinitiator (PI) may be required [3]. Most PIs form radicals either by homolytic cleavage or by hydrogen abstraction. Then, the radicals initiate the polymerization by reacting with the monomers. The most commonly used monomers in radical photopolymerization are acrylates and methacrylates. In search of sustainability, it is crucial to replace oil-based monomers with bio-based ones produced from renewable sources. Among the available biobased building blocks, some natural molecules can undergo autooxidation reactions, cyclization, isomerization, dimerization, and oligomerization in the presence of light [11,12]. However, most of these require being suitably functionalized prior to photoinduced polymerization processes. The introduction of polymerizable functions on biobased building blocks is thus a crucial step.

Recently, the use of naturally occurring phenols, such as eugenol and eugenol-derivatives, has gained attention for producing biobased monomers, as they can be obtained by lignin depolymerization [13,14,15]. In addition, eugenol-derived monomers are attractive because they possess antioxidant, antiseptic, and antibacterial properties [16,17], which could be exploited in photopolymers for dentistry or food packaging [18]. In particular, isoeugenol has a higher antibacterial activity than eugenol and is not genotoxic [19]. However, as many other biobased building blocks, eugenol and its derivatives do not possess functional groups that react readily through photoinduced polymerization. In addition, phenols scavenge free radicals and inhibit polymerization [20,21]. Thus, suitable functional groups must be inserted to avoid this inhibition and promote polymerization.

Besides the functionalization of eugenol with epoxy groups for cationic photopolymerization [22,23,24], methacrylate functional groups have also been introduced in the molecule. By reacting to the allylic double bond with 3-mercaptopropionic acid and thiomalic acid (*via* thiol-ene chemistry) and then reacting to the resulting carboxylic acid product and phenol group of eugenol with glycidyl methacrylate, eugenol methacrylic derivatives were obtained. These monomers were then used in photoinduced copolymerization with AESO (acrylated epoxidized soybean oil) to produce biobased coatings [25]. Moreover, allyl-etherified eugenol-derivatives were copolymerized through thiol-ene reactions with pentaerythritol-based primary and secondary tetrathiol and with isocyanurate-based secondary trithiol, to prepare crosslinked polymers [26]. Similarly, allyl-etherified eugenol and linalool were copolymerized with trimethylolpropane tris(3-mercaptopropionate) to form crosslinked networks endowed with antioxidant and antibacterial properties [18]. Later, a trifunctional allyl compound, tris(4-allyl-2 methoxyphenolyl) phosphate, was synthesized and reacted with thiols with two to four functionalities via thiol-ene chemistry and the influence of crosslink density on the different materials was studied [27]. Thiol-ene chemistry was also employed to covalently attach eugenol through its allylic double bond to a limonene-derived polymer network and prepare antibacterial coatings [28].

**Figure 1:**
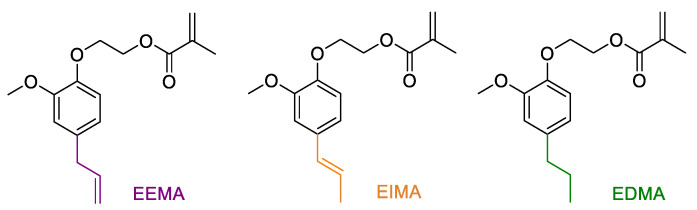
Eugenol-derived methacrylates.

An easy two-step synthetic alternative to produce methacrylated eugenol derivatives was described in a previous work [29]. A methacrylate group was introduced in eugenol structures taking advantage of its phenol group. Ethoxy eugenyl methacrylate (EEMA), ethoxy isoeugenyl methacrylate (EIMA) and ethoxy dihydroeugenyl methacrylate (EDMA) (Figure 1) were synthesized and successfully employed in solution and emulsion polymerization [29,30].

Biobased polymers obtained in the form of homogeneous and transparent films are potentially interesting for industrial development and could find application in coatings, food packaging or dentistry. Therefore, in the present article, we investigate the photopolymerization of films of these eugenol methacrylates under irradiation in different conditions: with or without the radical photoinitiator and in the presence or in the absence of air. Moreover, the conversion of the methacrylic double bond of the three monomers as well as the conversion of the allylic (EEMA) or propenyl (EIMA) double bonds are monitored, and the properties of the polymers are tested.

## 2. Results and Discussion

### 2.1. Kinetic Monitoring of Photoinduced Polymerization of Eugenol-Derived Methacrylates

The photoinduced polymerization of ethoxy eugenyl methacrylate (EEMA), ethoxy isoeugenyl methacrylate (EIMA) and ethoxy dihydroeugenyl methacrylate (EDMA) was conducted by irradiating the monomers spread on a solid substrate in the form of films. Different experimental conditions were investigated. At first, the reactions were attempted in the absence of any photoinitiator (PI). Avoiding the use of PI is a crucial step in the development of new products for many real life applications (e.g., inks for food packaging, dental materials) as photoinitiators decompose into harmful species which can uncontrollably migrate [31]. Then, reactions were done in the presence of two different Norrish Type I photoinitiators. Azo-initiators, largely used in radical polymerization, can also be used as photoinitiators. However, they have been reported to have low efficiency compared to acyl photoinitiators [32]. Thus, Darocur 1173 and Irgacure 819 have been selected. As the reaction proceeds via a radical mechanism, the effect of oxygen was studied by irradiating the monomers either in the presence or absence of air. Experiments in the absence of air were carried out by covering the monomer films with a polypropylene (PP) film. This is a common strategy to protect polymerization samples from oxygen and reduce inhibition [33].

#### 2.1.1. Photopolymerization Without Photoinitiator

**Figure 2:**
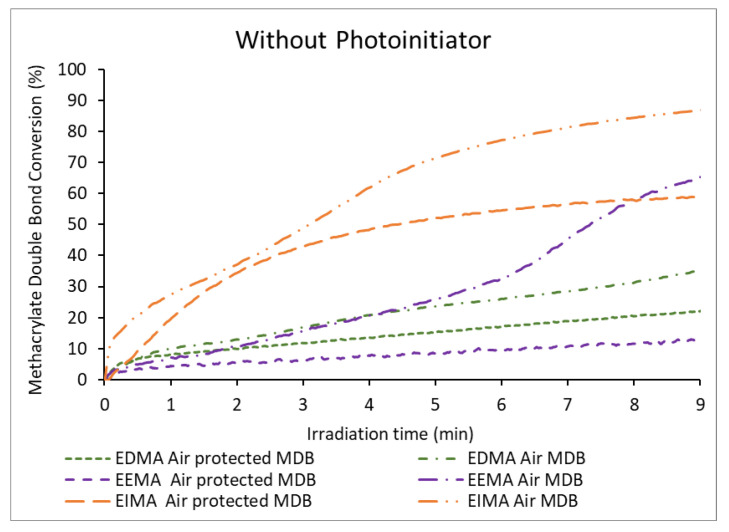
Methacrylate double bond (MDB) conversion of eugenol-derived monomers versus irradiation time in the absence of photoinitiator.

**Figure 3:**
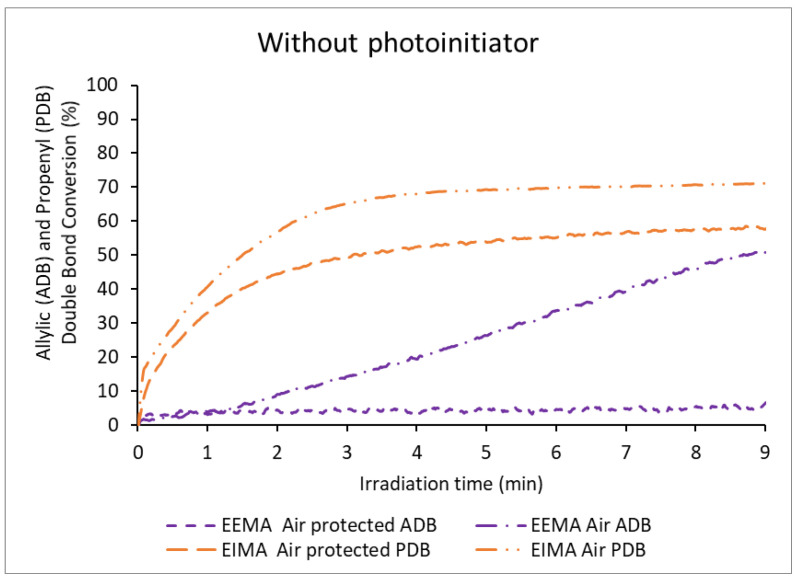
Allylic (ADB) and Propenyl double bond (PDB) conversion of eugenol-derived monomers versus irradiation time in the absence of photoinitiator.

The kinetics of the reactions of the eugenol-derivatives EDMA, EEMA, and EIMA were monitored by Real-Time FT-IR in transmission mode while they were exposed to a UV-light source (L9566-02A, 240 to 400 nm, 260 mW cm^−2^) [34] in the presence of air or in the absence of air. The band corresponding to the methacrylate double bond at 1638 cm^−1^ (C=C stretching vibration) [35], and the aromatic band at 1514 cm^−1^ (C-H aromatic in-plane bending) [36] as reference, were monitored over the irradiation time. The conversion of the methacrylate double bonds (MDB) for EDMA, EEMA, and EIMA are presented in Figure 2. Simultaneously, the conversion of the allylic double bonds (ADB) from EEMA and propenyl double bonds (PDB) from EIMA were monitored using the bands at 995 cm^−1^ and 960 cm^−1^ respectively. The results comparing allylic and propenyl double bond conversion with regards to the presence or absence of air are plotted in Figure 3.

Figure 2 shows that the methacrylic double bonds (MDB) of all monomers can react upon light exposure even in the absence of any photoinitiator. This is not surprising, as it has been previously reported that (meth)acrylates could undergo photopolymerization without a photoinitiator due to self-initiation [37,38,39,40]. Interestingly, the final conversion of MDB and the conversion rate (i.e., the slope of the conversion versus time curve) were different for the three monomers. The reactivity trend is as follows: EIMA >> EDMA > EEMA. EIMA was the most reactive monomer (higher slope, final conversion of 59%), while the conversion of EDMA and EEMA remained low reaching only 22% and 12% respectively at the end of the irradiation (Table 1). Conversion rates of EDMA and EEMA were much lower than that of EIMA. The different reactivities of the MDBs may be explained by the difference in the UV absorption spectra of the monomers (Figure S4). The monomers UV absorption curves overlap with the emission spectrum of the Hg lamp used as irradiation source [34]. The absorption of EIMA is significantly higher than that of EDMA and EEMA. EIMA is thus more likely to undergo faster self-initiation. To confirm that the monomers self-initiate due to UV absorption [37], further polymerization experiments protected from air were performed under UV irradiation but using a filter to stop wavelengths below 365 nm. As expected, since the monomers absorb below 320 nm, no reaction was observed for any of the monomers. These experiments confirmed the hypothesis of self-initiation responsible for the polymerization occurring in the absence of PI.

**Figure 4:**
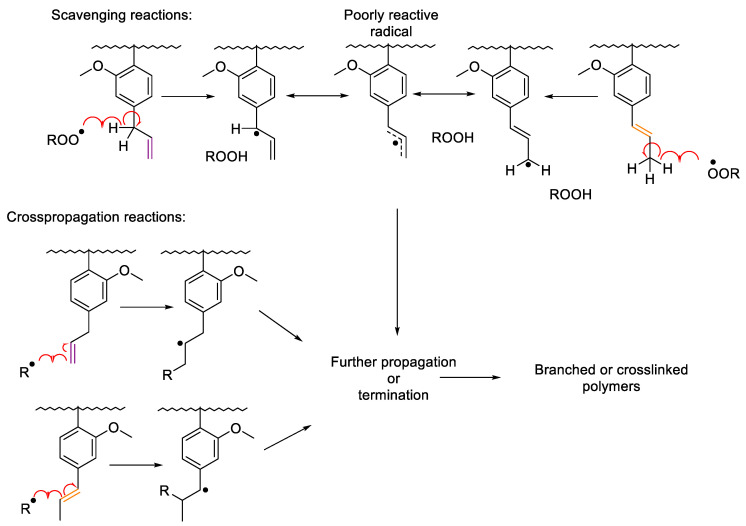
Scavenging and cross-propagation reactions on allylic and propenyl double bonds of eugenol-derived methacrylates.

Besides the methacrylic group, the reactivity of the allyl and propenyl groups, present in EEMA and EIMA respectively, were studied during photopolymerization processes protected from air (Figure 3). Indeed, allylic and propenyl double bonds can experience secondary reactions such as (degradative) chain transfer reactions (allylic hydrogen abstraction produce poorly-reactive highly-stabilized radicals) and radical addition (cross-propagation) (Figure 4) [29,41,42,43]. The radicals formed from these secondary reactions can undergo further propagation or termination yielding branched and even crosslinked polymers (in the case of termination by combination). In the absence of air, the propenyl double bond (PDB) of EIMA was quite reactive and reached nearly the same conversion as the methacrylic double bonds (58%). On the other hand, the allylic double bond (ADB) of EEMA displayed a very low conversion (6%). For this monomer, a lower reaction rate and MDB conversion were obtained (Figure 2). EEMA allylic hydrogens can be abstracted and form highly stabilized radicals (main secondary reaction). This affects the propagation rate as the corresponding radicals become less reactive (degradative chain transfer). This effect was not seen for EIMA, implying that PDB reacts mainly though cross-propagation reactions between propenyl and methacrylic groups.

Figure 2 shows that the methacrylate double bond conversions under air have different profiles compared to those of the polymerization protected from air. Changes in the curve slope are visible, signaling a noticeable variation of the speed of the reaction during the irradiation. This behavior is particularly clear for EIMA and to a lower extent for EEMA (Figure 2), but it is negligible for EDMA. The sigmoidal profile appearing in the curves is caused by the occurrence of two polymerization regimes. These are due to the formation of hydroperoxides in the presence of oxygen, as reported in literature [44]. Indeed, radicals produced by irradiation of the monomers can react with oxygen according to Equations (1)–(3).

(1) $$R^{\cdot }+O_{2}\to RO_{2}{}^{\cdot }$$

(2) $$\;\;RO_{2}{}^{\cdot }+RH\to \;RO_{2}H+R^{\cdot }\;\;\;\;$$

(3) $$\;\;RO_{2}H\overset{hv\;}{\to }RO^{\cdot }+HO^{\cdot }\;\;$$

Once peroxyl radicals (RO_2_) are formed (Equation (1)), hydroperoxides (RO_2_H) are generated by hydrogen abstraction (Equation (2)) [33]. The three monomers possess abstractable hydrogen atoms: bis-allylic hydrogens in EEMA (Ph- C**H_2_**- CH= CH_2_), propenylic hydrogens in EIMA (Ph- CH= CH- C**H_3_**), and benzylic hydrogens in EDMA (Ph- C**H_2_**- CH_2_- CH_3_). Hydroperoxides react slowly, therefore oxygen is often described as an inhibitor of radical polymerization. The phenomenon is particularly severe in photopolymerization when monomers are irradiated in films. In such a situation, a large area is exposed to oxygen, and oxygen can be continuously replaced by diffusion at the surface of the reacting formulation. However, hydroperoxides can decompose, through continued irradiation, to produce new radicals (Equation (3)) that are able to trigger additional initiation and a second polymerization regime [44]. Herein, IR analyses confirmed the hydroperoxides formation during the photopolymerization reactions carried out in the presence of air (Figures S12–S14).

Contrary to what was expected, all the monomers showed a higher MDB conversion in the presence of air than in the absence of air. During the first minute of irradiation, EEMA and EDMA displayed very similar MDB conversion under air or protected from air. However, conversion increased significantly at higher irradiation time in the presence of air. Specifically, for EEMA, MDB final conversion reached 66% under air (and only 12% when protected from air over the same irradiation time). For EDMA, the final conversion was 35% in the presence of air and 22% when protected from air (Table 1). Finally, for EIMA, the MDB conversion and conversion rates under air were always higher than in the absence of air from the onset of irradiation. Similarly to EEMA, after the first polymerization regime, the conversion rate of EIMA MDB increased (producing a second polymerization regime) and a final conversion of 86% was reached (59% in the absence of air).

Figure 3 shows that EEMA ADB are consumed up to 49% in the presence of air, while they are almost non-reactive in the absence of air. This can explain the high reactivity of the EEMA MDBs under air. Allylic double bonds undergo hydrogen abstraction leading to radicals that can react with oxygen to form peroxy radicals which scavenge oxygen and thus prevent the oxygen inhibition of MDB polymerization. The peroxy radicals can then form hydroperoxides that decompose to provide additional radicals for further MDB polymerization. In the case of EIMA, the conversion of PDB reached relatively high values both under air (68%) and while air-protected (58%) and was always higher under air, independent of the irradiation time (Figure 3 and Table 1). PDB can be consumed not only by the formation of peroxy and hydroperoxy radicals when oxygen is present but also by cross-propagation reactions (Figure 4). Noteworthy is that only a slightly higher consumption of PDB was observed in the polymerizations carried out under air compared to the polymerizations protected from it. This suggests that although hydroperoxides are formed under air, cross-propagation is the main secondary reaction.

In addition, higher conversion of the monomers under air can also be related to the formation of ozone induced by the UV irradiation at 242 nm and its subsequent photolysis into singlet oxygen (^1^O_2_) [45,46]. Singlet oxygen can react with the ADB and PDB of both EEMA and EIMA, again forming peroxy radicals and hydroperoxides which dissociate into other radicals [47,48,49,50].

Styrene can polymerize in the absence of a photoinitiator, due its capacity to form charge-transfer complexes with oxygen [51,52]. These complexes lead to the production of peroxides eventually leading to the production of radicals. Recently, Krueger et al. [53] concluded that in photoinitiator-free styrene polymerizations, oxygen reacts photochemically with styrene at the beginning of their polymerization reactions but that peroxides are not the sole source of radical formation. The photochemical radical generation via photo electron transfer (PET) requires a donor-acceptor pair. In the absence of oxygen, the PET between styrene-polystyrene leads to the generation of radical ions continuing the polymerization in the absence of PI. A similar process could occur in the case of isoeugenol. Moreover, the triplet state of isoeugenol derivatives has been suspected to produce singlet oxygen able to react with the double bond to form dioxetane, which can cleave to produce aldehydes [52,54]. Nonetheless, no increment or change was noticed in the bands at 2827 cm^−1^ and 2725 cm^−1^, corresponding to the Fermi resonance characteristic of aldehydes. Hence, the consumption of PDB does not follow this pathway here.

To avoid the absorption of light by the monomers and the possible formation of ozone, further polymerization experiments under air were performed using a 355–375 nm bandpass filter. Unsurprisingly, no reaction was observed for any of the monomers, since neither monomer homolytic cleavage nor ozone production (both leading to radicals) occur at this longer irradiation wavelength.

In conclusion, the eugenol-derived methacrylates can photopolymerize in the absence of the photoinitiator both in the presence or absence of air as long as the irradiation wavelengths are short (from 220 to 355 nm). The presence of oxygen (while irradiating at short wavelengths <365 nm) leads to higher conversion of the methacrylic, allylic, and propenyl double bonds of the eugenol-derived methacrylates as a consequence of the production of hydroperoxides and their decomposition. The presence of ADB and PDB causes secondary reactions such as allylic hydrogen abstraction and cross-propagation which could lead to branched or crosslinked structures.

#### 2.1.2. Photopolymerization with Photoinitiator

**Figure 5:**
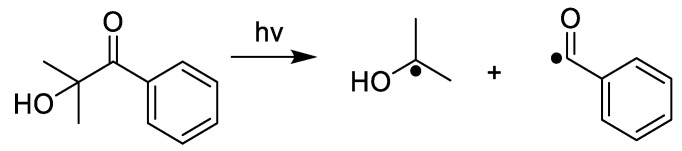
Homolytic cleavage under light of Darocur 1173.

Experiments proceeded with the use of common photoinitiators. Darocur 1173 was added to the monomers at 2% wbm (weight based on monomer). It is a Norrish Type I photoinitiator that undergoes homolytic cleavage to produce two carbon-centered radicals (Figure 5 and Figure S5).

**Figure 6:**
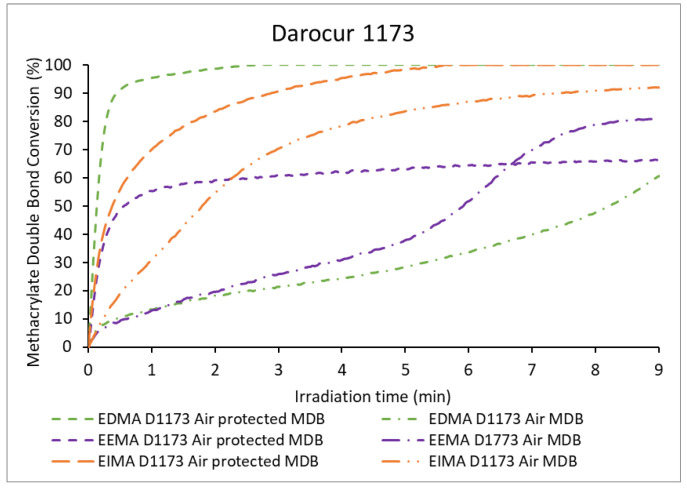
Methacrylate double bond (MDB) conversion of eugenol-derived monomers versus irradiation time in the presence of Darocur 1173.

**Figure 7:**
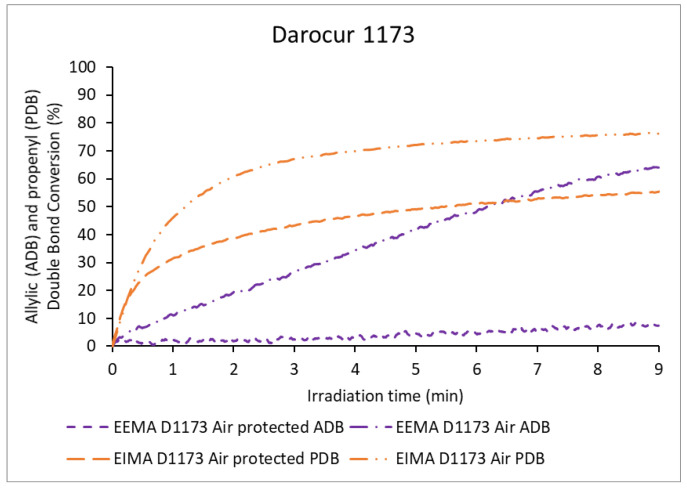
Allylic (ADB) and propenyl double bond (PDB) conversion of eugenol-derived monomers versus irradiation time in the presence of Darocur 1173.

The evolutions with irradiation time of the MDB conversions of the three monomers for the photopolymerizations carried out in the presence and in the absence of air using Darocur 1173 are shown in Figure 6. The evolutions with time of the conversions of ADB and PDB in the same conditions are displayed in Figure 7. The comparison of these data with those observed for polymerizations carried out in the absence of the photoinitiator demonstrates, as expected, that the PI accelerates the polymerization.

In the absence of air, the conversion of EDMA MDB was fast and reached 100%. The polymerization rates of the difunctional methacrylates, EEMA and EIMA, were slower. EEMA MDB conversion reached 66%, whereas that of EIMA MDB reached 100% although at a lower rate than EDMA (Table 1). In the case of EEMA, radicals were presumed to be consumed by the allyl groups (degradative chain transfer), even to a small extent, to form highly stabilized radicals that resulted in a lower polymerization rate and ultimately in termination reactions limiting the conversion. No increment or appearance of the band at 960 cm^−1^ corresponding to the propenyl double bonds was observed, suggesting that the isomerization of EEMA into EIMA does not occur under these experimental conditions. In addition, the conversion of allylic double bonds to propenyl double bonds would lead to the decrease of the 995 cm^−1^ peak area (corresponding to allylic double bond), which did not occur, as conversion was <10%. In the case of EIMA, the PDBs were consumed up to 56% (Table 1) most likely via cross-propagation (vide supra). This cross-propagation slightly slows down the polymerization but does not prevent the quantitative conversion of EIMA MDB. Moreover, as discussed above, EIMA has a higher absorption than EEMA and forms propagating species by itself, thus enhancing the conversion.

In the presence of air, the polymerization rates were lower than those observed for polymerizations carried out in the absence of air. This decrease of the polymerization rate was likely caused by oxygen inhibition. EDMA was strongly inhibited by air and presented the lowest MDB conversion (61%). EIMA and EEMA were less affected and reached high conversions: 92% and 81%, respectively (Table 1). As previously discussed, reactions with oxygen can lead to the formation of hydroperoxides, which decompose, causing a second polymerization regime. The corresponding sigmoidal curve is observed quite clearly for EEMA, but not for EIMA. In addition, the conversion of PDB was higher than that of ADB (76% and 64% respectively, Figure 7). PDBs were also highly consumed when protected from air (56%), while ADBs were not (7%). This may be because cross-propagation is the dominant reaction in the consumption of PDBs (only a fraction might be consumed by hydrogen abstraction or hydroperoxide formation). In contrast, in the case of EEMA, for which bis-allylic H-abstraction and radical termination dominate, the absence of air limits the overall polymerization. However, in the presence of air, hydroperoxide dissociation provides the necessary radicals to continue EEMA MDB polymerization.

**Figure 8:**
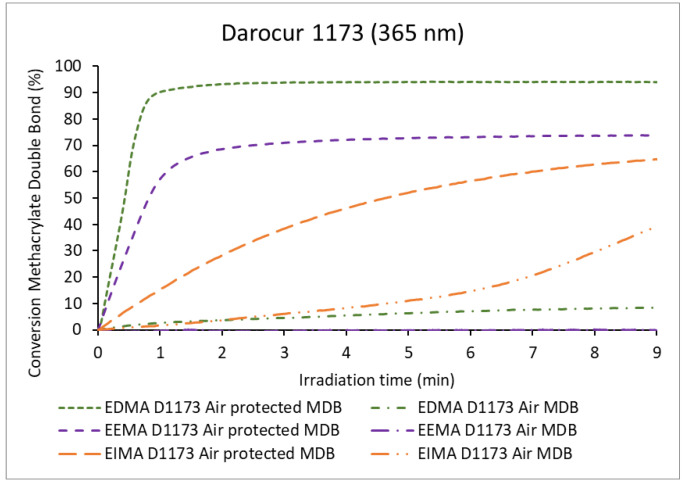
Methacrylate double bond (MDB) conversion of eugenol-derived monomers with irradiation time in the presence of Darocur 1173, irradiation under λ = 365 nm.

**Figure 9:**
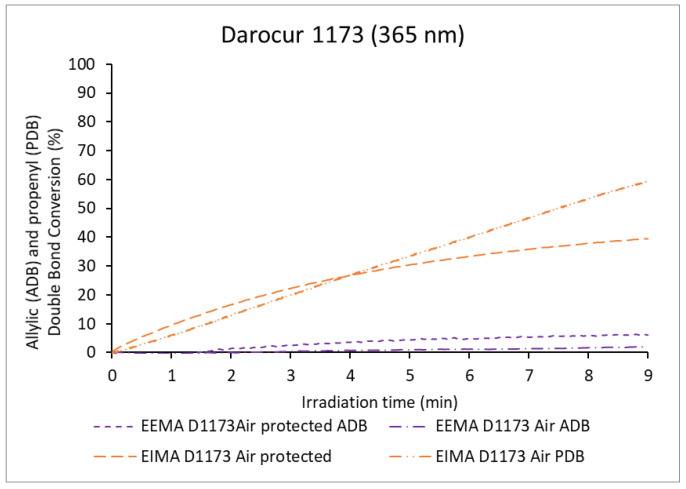
Allylic (ADB) and propenyl double bond (PDB) conversion of eugenol-derived monomers with irradiation time in the presence of Darocur 1173, irradiation under λ = 365 nm.

The effect of the monomer light absorption and the possible formation of ozone on the kinetics of polymerization remained to be investigated. Thus, a bandpass filter centered at 365 nm, preventing monomer light absorption and ozone formation (<242 nm) was again used to irradiate the formulations. Experiments with Darocur 1173 were performed and results are shown in Figure 8 and Figure 9.

In the presence of air, almost no conversion of MDB could be measured for EEMA and EDMA (conversion <10%, Figure 8 and Table 1). Similar results were observed for EEMA ADB with a conversion close to zero (2%, Figure 9 and Table 1). In the presence of the filter, the production of radicals by cleavage of the photoinitiator was strongly diminished and the scarce quantity of radicals could quickly be quenched by oxygen, while no peroxides nor hydroperoxides could be generated [33]. Nevertheless, the considerable consumption of both EIMA MDB and PDB (39% and 58% respectively, Table 1 was observed in spite of the presence of oxygen. This may be explained by the formation of charge-transfer complexes of EIMA with oxygen (as reported for styrene [51,52]) which lead to the production of radicals and allow propagation.

In the absence of air, both the polymerization rate and the final conversion increased significantly. MDB conversion followed the trend: EDMA (94%) > EEMA (74%) > EIMA (65%) (Table 1). The polymerization rate was lower than that observed for the reaction carried out using light, including shorter irradiation wavelengths (i.e., without filter). This may be explained by a lower radical production both from Darocur 1173 (which absorbs weakly at 365 nm) and from the monomers (which do not absorb at 365, see Figure S4). Moreover, the irradiance decreases because of the filter (78 mW.cm^−2^ UVA). However, contrary to the experiments carried out without filter, EIMA showed lower MDB conversion than EEMA. This means that EIMA UV light absorption and cleavage (responsible for the reaction in the absence of PI) contribute to the formation of reactive species. Moreover, the consumption of the PDBs reached 40% (Table 1), while ADB consumption remained low (6%).

The comparison of the results of the polymerizations irradiated with and without filter using Darocur 1173 (Norrish Type I photoinitiator) suggests that the monomer absorptions play an important role in their reactivity, especially for EIMA. Moreover, in the presence of air, reactions of peroxides and hydroperoxides, ozone formation and photolysis to singlet oxygen, contribute to the polymerization mechanism.

In a final study, a passband filter centered at 365 nm and another Norrish type I PI with high absorption at longer wavelengths (absorption in the UVA region), Irgacure 819 [55] were used. The results obtained in the different conditions (with and without air) are gathered in the SI (Figures S16 and S17). In this case, the potential cleavage of the methacrylates was prevented by the pass band filter and oxygen inhibition or the production of hydroperoxides was avoided by protecting the samples from air. In addition, the flux of radicals had been raised by using Irgacure 819, which has a higher molar extinction coefficient and quantum yield than Darocur 1173 in the UVA region [55]. A behavior similar to that of Darocur 1173 was observed.

The experiments executed in different conditions (with or without initiator, in the presence and absence of air, with or without filter) revealed that EDMA polymerization was always strongly inhibited in the presence of air. On the contrary, the presence of the pending allylic (EEMA) or propenyl (EIMA) double bonds could produce a second polymerization regime due to dissociation of hydroperoxides (formed in-situ in the presence of air under shorter (<320 nm) wavelength irradiation). It was also shown that the dominant reaction mechanism for PDB is cross-propagation rather than hydrogen abstraction or hydroperoxide formation, as they were consumed to a high extent even in the absence of air. The polymerization of EIMA was the least affected by air.

### 2.2. Polymers Characterization

Properties of the polymers prepared by photoinduced polymerization in the presence of Darocur 1173, both in the presence and absence of air, were measured (Table 2). The polymerization conditions (i.e., use of a UV irradiation spectrum from 320 to 390 nm and of Darocur 1173 as PI) were selected to guarantee high conversions. Polymers obtained from EDMA had a linear structure and were soluble (gel content ≈ 0%). In contrast, polymers from EEMA and EIMA were crosslinked and completely insoluble (gel content = 100%), suggesting that the unreacted functional groups potentially present (when the conversion was not quantitative, as reported in Table S2) were dangling from the network and that no free oligomer or monomer were present. The glass transition temperature did not vary much between the samples irradiated in the presence or in the absence of air except for poly(EDMA) prepared by irradiation under air. In this case, the presence of oligomers or unreacted monomer plasticized the resulting polymer and reduced its *T*_g_. The obtained *T*_g_s were in agreement with the results previously obtained with polymers prepared by emulsion polymerization [30].

**Figure 10:**
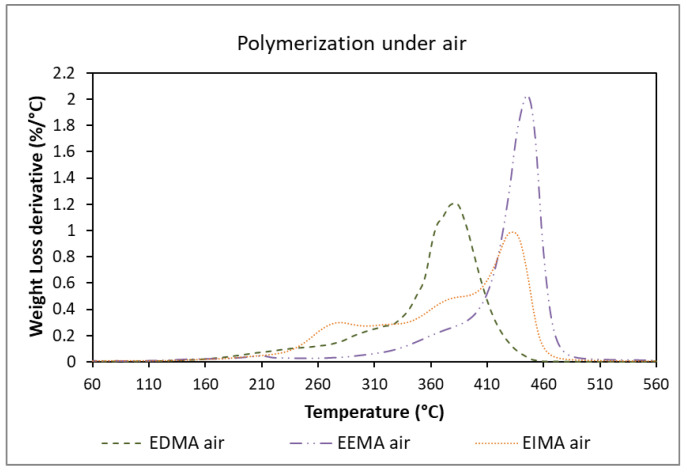
Thermogravimetric analysis of the different eugenol-derived methacrylates polymers from polymerization with Darocur 1173 under air (1° derivative).

**Figure 11:**
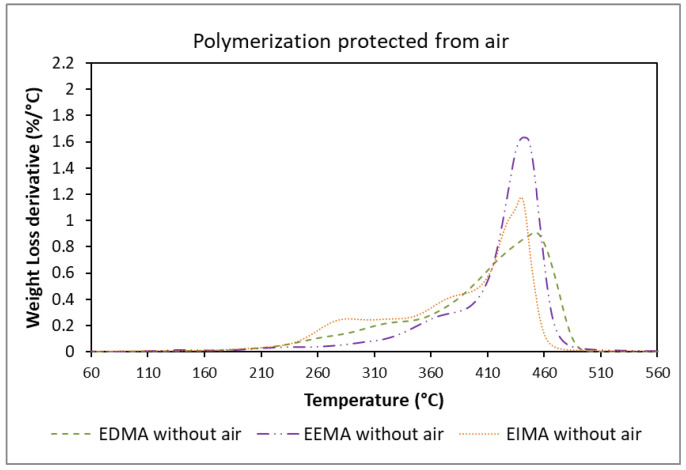
Thermogravimetric analysis of the different eugenol-derived methacrylates polymers from polymerization with Darocur 1173 protected from air (1° derivative).

The TGA results showed that the starting degradation temperatures of the polymers were always higher than 230 °C (Figure 10 and Figure 11). Polymerization carried out in the presence of air led to crosslinked poly(EEMA) with higher decomposition temperatures, due to a higher consumption of ADBs. A slightly lower degradation temperature was registered for poly(EIMA) prepared in the presence of air but both polymers (produced under air or in the absence of air) exhibited complex profiles, indicating complex polymeric architectures. Their glass transition temperatures (ranging from 8 °C and 58 °C) as well as their degradation temperatures (above 230 °C) make these materials suitable for application in coatings.

The water and hexadecane contact angles (Table 2) indicated that the wettability of all the polymers were independent of the structure. The polymers were almost hydrophobic and displayed moderate oleophilicity.

## 3. Materials and Methods

### 3.1. Materials

**Figure 12:**
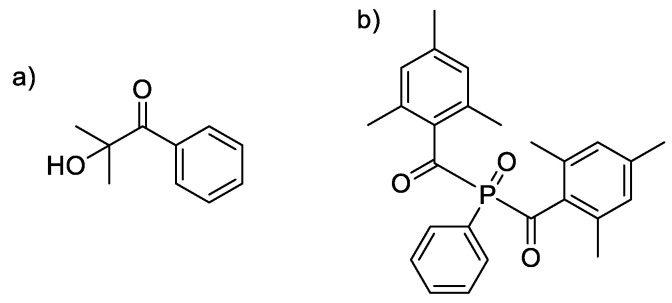
Photoinitiators (**a**) Darocur 1173 (**b**) Irgacure 819.

Ethoxy eugenyl methacrylate (EEMA), ethoxy isoeugenyl methacrylate (EIMA), ethoxy dihydroeugenyl methacrylate (EDMA) monomers (Figure 1) were synthesized as described in a previous article from our group [29]. Toluene (>98%) was purchased from Sigma-Aldrich (Milano, Italy). The radical photoinitiators 2-hydroxy-2-methylpropiophenone (tradename Darocur 1173) and phenylbis(2,4,6-trimethylbenzoyl)phosphine oxide (tradename Irgacure 819), kindly given by Badische Anilin und Soda Fabrik (BASF) (Ludwigshafen, Germany) were used as received. Darocur 1173 and Irgacure 819 structures are presented in Figure 12.

### 3.2. Photoinduced Polymerization of Eugenol Derived Methacrylates

#### 3.2.1. Samples Preparation

To monitor the photopolymerization kinetics of each monomer, a mixture of monomer and PI at 2% wbm (weight based on monomer) was spread over a silicon wafer using a rod coater, forming a film with a thickness of 10 µm. Samples were irradiated up to 9 min either under air or protected from air with a 30 μm-thick polypropylene (PP) film.

#### 3.2.2. Kinetics Monitoring

Photopolymerization was monitored using Real-Time Fourier Transform Infrared (FT-IR) spectroscopy on a Nicolet iS50 spectrometer (Thermo Fisher Scientific Inc., Waltham, MA, USA). The spectra were acquired in transmission mode, in the 650–4000 cm^−1^ range, with 1 scan per spectrum and a resolution of 4 cm^−1^.

A high-pressure mercury-xenon lamp Lightning Cure LC8 from Hamamatsu equipped with a flexible light guide was used as UV-light source (L9566-02A, 220 to 600 nm) [34] and an EIT Powerpuck^®^ II radiometer (EIT, LLC., Leesburg, VA, USA) was used to measure the UV irradiance. The samples were irradiated with 260 mW cm^−2^ (sum of UVA, UVB, UVC, UVV). In some experiments, light was filtered using a A9616-07 filter (Hamamatsu, Shizuoka, Japan) with a transmittance wavelength of 355–375 nm (centered at 365 nm) The filtered light had and intensity of 78 mW cm^−2^ (UVA).

#### 3.2.3. Conversion Determination

The methacrylate double bonds (MDB) conversion was monitored using the band at 1638 cm^−1^, the allylic double bonds (ADB) conversion was monitored using the band at 995 cm^−1^, and the propenyl double bonds (PDB) conversion was determined using the 960 cm^−1^ band (Figure S1) [26,36,56,57]. Each conversion was calculated using the following Equation (4):

(4) $$Conversion{\%}_{t=x}=100\times \left(1-\frac{\frac{A_{t=x}}{Ref.A_{t=x}}}{\frac{A_{t=0}}{Ref.A_{t=0}}}\right)$$

Where A is the absorbance of the IR band of the functional group monitored during irradiation; Ref A is the absorbance of the band of the aromatic ring (C-C stretching) taken as a reference (1540 cm^−1^ to 1490 cm^−1^).

Absorbances were estimated as the area of the vibrational bands under examination. Data were processed using OMNIC software (Madison, WI, USA). All curves were smoothed using the Savitzky–Golay method with 20 points window and second polynomial order. For the determination of EEMA methacrylate double bonds conversion, an approximation was made as peaks corresponding to the allylic and methacrylate double bond superimposed at circa 1638 cm^−1^. The calculation details are explained in SI (Figures S2 and S3 and Table S1).

### 3.3. Characterization Methods

Samples for thermogravimetric analysis (TGA), differential scanning calorimetry (DSC), and gel content were prepared by coating a glass slide with 200 µm films and irradiating it for 10 min using a DYMAX 5000 EC UV flood lamp (Dymax Corporation, Torrington, CT, USA) in the range of 320 to 390 nm with an intensity on the sample of 156 mW cm^−2^ (UVA and UVV).

IR spectra to determine the conversion were acquired on a Thermo Scientific Nicolet 6700 FTIR apparatus (Madison, WI, USA) in the 650–4000 cm^−1^ range, with 32 scan per spectrum and a resolution of 4 cm^−1^ (using attenuated total reflectance technique, ATR) on both faces of the film: the one exposed to the atmosphere and the one in contact with the glass slide.

Ultraviolet and visible spectroscopy (Figures S4 and S5): UV-Vis spectra were recorded on a Varian Cary 50 Scan spectrophotometer (Victoria, Australia) from 200 to 800 nm with a scan rate of 4800 nm min^−1^.

Thermogravimetric Analysis: TGA was performed on 5–10 mg samples on a TGA Q50 apparatus from TA Instruments (New Castle, DE, USA) from 20 °C to 580 °C, in an aluminum pan, at a heating rate of 20 °C min^−1^, under nitrogen.

Differential Scanning Calorimetry: (Figures S6–S11) DSC measurements were performed on 10–15 mg samples, under nitrogen atmosphere, with a Netzsch DSC 200 F3 instrument (Selb, Germany) using the following heating/cooling cycle: first cooling ramp from room temperature (ca. 20 °C) to −40 °C at 20 °C min^−1^, isothermal plateau at −40 °C for 10 min, first heating ramp from −40 to 150 °C at 20 °C min^−1^, second cooling stage from 150 to −40 °C at 20 °C min^−1^, isothermal plateau at −40 °C for 10 min, second heating ramp from −40 to 150 °C at 20 °C min^−1^, third cooling stage from 150 to −40 °C at 20 °C min^−1^, isothermal plateau at −40 °C for 10 min, third heating ramp from −40 to 150 °C at 20 °C min^−1^ and last cooling stage from 150 °C to room temperature (ca. 20 °C). *T_g_* values are given from the evaluation of the third heating ramp. Calibration of the instrument was performed with noble metals and checked with an indium sample.

Gel content measurements: The gel content of the polymers was measured by placing approximately 30–50 mg of polymer in a Teflon pocket which was subsequently immersed in 10 mL of toluene for 24 h, then dried in a ventilated oven at 50 °C for 4 h. The gel content was calculated based on the initial (*W_i_*) and final (*W_f_*) polymer mass according to Equation (5).

(5) $$Gel\;content\left(\%\right)=\frac{W_{f}\times 100}{W_{i}}$$

## 4. Conclusions

Three eugenol-derived methacrylates (EDMA, EEMA, EIMA) were polymerized via photopolymerization without a photoinitiator and with two Norrish Type I photoinitiators (Darocur 1173 and Irgacure 819), under air or without air. Their polymerization behavior under the different conditions was described. The monomers were shown to polymerize in the absence of a photoinitiator, especially in the presence of oxygen, due to self-initiation and oxidation reactions. In the presence of air, EIMA showed the highest conversions in any of the conditions studied. The second polymerization regimes, due to the formation and photolysis of hydroperoxides, were observed upon irradiation at a wavelength shorter than 365 nm in air with or without PI. This effect was clearly visible for EIMA and EEMA. Moreover, both allylic and propenyl double bonds were reactive in the presence of air. In addition, PDBs were shown to be predominantly polymerized via cross-propagation reactions while ADBs were mainly consumed under air via hydrogen abstraction and hydroperoxides formation. In the absence of air and using PI, EDMA reached the highest conversions. To eliminate the self-initiation of the monomers as well as the formation of hydroperoxides, a 365 nm passband filter and air-protected conditions were used. Under these conditions, the polymerization rate followed the order EDMA > EEMA > EIMA. EEMA displayed a significant reduction of the propagation rate, due to the formation of highly stabilized bis-allylic radicals. EIMA exhibited a lower MDB conversion, due to cross-propagation with the PDB. The polymers properties indicated that their use in applications in coatings and in dentistry could be envisaged.

## Figures and Tables

**Table 1:** Methacrylate (MDB), allylic (ADB) and propenyl (PDB) double bond conversions under different conditions of irradiation.

Monomer	Condition	Conversion (%)			
		220–600 nm		365 nm	
		Without PI	Darocur 1173	Darocur 1173	Irgacure 819
EDMA MDB	Air protected	22	100	94	96
EEMA MDB		12	66	74	76
EIMA MDB		59	100	65	78
EEMA ADB		6	7	6	3
EIMA PDB		58	56	40	12
EDMA MDB	Under air	35	61	8	8
EEMA MDB		66	81	0	7
EIMA MDB		86	92	39	40
EEMA ADB		49	64	2	9
EIMA PDB		68	76	58	30

**Table 2:** Thermal properties, gel content, and contact angle of homopolymers produced with Darocur 1173.

Monomer	Polymerization Condition	Gel Content (%)	*T*_g_ (°C)	*T*_d5%_ (°C)	Contact Angle DI Water (°)	Contact Angle Hexadecane (°)
EDMA	with air	2	8	236	92	24
	no air	3	23	269	84	30
EEMA	with air	100	35	298	89	33
	no air	98	34	294	85	34
EIMA	with air	100	56	246	85	24
	no air	100	58	258	82	25

## Supplementary Materials

The following are available online. Figure S1. IR spectra in transmission of the different eugenol derived methacrylates: (A) EDMA, (B) EEMA and (C) EIMA, Figure S2. Eugenol, ethoxy eugenol, and ethoxy eugenyl methacrylate, Table S1. Normalization of the allylic band area for Eugenol and EE, Figure S3. ATR Spectra of eugenol, ethoxyeugenol, and ethoxy eugenyl methacrylate, Figure S4. UV absorption spectra of the eugenol-derived methacrylates, in acetonitrile 0.002 wt%, Figure S5. UV absorption of photoinitiators, Figure S6. DSC measurement of poly(EDMA) obtained from photoinduced polymerization with Darocur 1173 (2% wbm) under nitrogen, Figure S7. DSC measurement of poly(EDMA) obtained from photoinduced polymerization with Darocur 1173 (2% wbm) under air, Figure S8. DSC measurement of poly(EEMA) obtained from photoinduced polymerization with Darocur 1173 (2% wbm) under nitrogen, Figure S9. DSC measurement of poly(EEMA) obtained from photoinduced polymerization with Darocur 1173 (2% wbm) under air, Figure S10. DSC measurement of poly(EIMA) obtained from photoinduced polymerization with Darocur 1173 (2% wbm) under nitrogen, Figure S11. DSC measurement of poly(EIMA) obtained from photoinduced polymerization with Darocur 1173 (2% wbm) under air, Figure S12. FT-IR spectra at time = 0 and time = 9 min denoting the formation of hydroperoxides during photopolymerization of EEMA in the absence of PI under air, Figure S13. FT-IR spectra at time = 0 and time = 9 min denoting the formation of hydroperoxides during photopolymerization of EIMA in the absence of PI under air, Figure S14. FT-IR spectra at time = 0 and time = 9 min denoting the formation of hydroperoxides during photopolymerization of EDMA in the absence of PI under air, Figure S15. Irgacure 819, its homolytic cleavage under light, Figure S16. Methacrylate double bond conversion for eugenol-derived monomer versus irradiation time with Irgacure 819 and filter (dotted lines = irradiation under air; solid lines = irradiation with air protection), Figure S17. Allylic and propenyl double bond conversion for eugenol-derived monomers versus irradiation time with Irgacure 819 and filter (dotted lines = irradiation under air; solid lines = irradiation with air protection), Table S2. Monomer conversion of films used for polymer characterization (by ATR-FTIR).
