# Supplementary material for: Photoinduced Polymerization of Eugenol-Derived Methacrylates
Source: Molecules. 2020 Jul 29;25(15):3444. doi: 10.3390/molecules25153444 (PMC7435665; doi:10.3390/molecules25153444)
Supplement: Supplementary file 1 [file molecules-25-03444-s001.pdf]

Supporting Information for

Photoinduced polymerization of eugenol-derived methacrylates

Samantha Molina-Gutiérrez,[1,2] Sara Dalle Vacche,[2] Alessandra Vitale,[2] Vincent Ladmiral,[1] Sylvain Caillol,[1] Roberta Bongiovanni,[2,*] and Patrick Lacroix-Desmazes[1,*]

[1] ICGM, Univ Montpellier, CNRS, ENSCM, Montpellier, France; samantha.molina-gutierrez@enscm.fr (S.M.G), vincent.ladmiral@enscm.fr (V.L.), sylvain.caillol@enscm.fr (S.C)

[2] Department of Applied Science and Technology, Politecnico di Torino, Corso Duca degli Abruzzi 24, 10129 Torino, Italy; sara.dallevacche@polito.it (S.D.V), alessandra.vitale@polito.it (A.V.)

* Correspondence: roberta.bongiovanni@polito.it (R.B.), patrick.lacroix-desmazes@enscm.fr (P.L.D.)

Table of Figures

Figure S1. IR spectra in transmission of the different eugenol derived methacrylates: A) EDMA, B) EEMA and C) EIMA S2
Figure S2. Eugenol, ethoxy eugenol and ethoxy eugenyl methacrylate S3
Figure S3. Transmission spectra of eugenol, ethoxyeugenol and ethoxy eugenyl methacrylate. S3
Figure S4. UV absorption spectra of the eugenol-derived methacrylates, in acetonitrile 0.002 wt%. S5
Figure S5. UV Absorption of photoinitiators. S5
Figure S6. DSC measurement of poly(EDMA) obtained from photoinduced polymerization with Darocur 1173 (2% wbm) under nitrogen. S6
Figure S7. DSC measurement of poly(EDMA) obtained from photoinduced polymerization with Darocur 1173 (2% wbm) under air. S6
Figure S8. DSC measurement of poly(EEMA) obtained from photoinduced polymerization with Darocur 1173 (2% wbm) under nitrogen. S7
Figure S9. DSC measurement of poly(EEMA) obtained from photoinduced polymerization with Darocur 1173 (2% wbm) under air. S7
Figure S10. DSC measurement of poly(EIMA) obtained from photoinduced polymerization with Darocur 1173 (2% wbm) under nitrogen. S8
Figure S11. DSC measurement of poly(EIMA) obtained from photoinduced polymerization with Darocur 1173 (2% wbm) under air. S8
Figure S12. FT-IR spectra at time=0 and time=9 minutes denoting the formation of hydroperoxides during photopolymerization of EEMA in the absence of PI under air. S9
Figure S13. FT-IR spectra at time=0 and time=9 minutes denoting the formation of hydroperoxides during photopolymerization of EIMA in the absence of PI under air. S9
Figure S14. FT-IR spectra at time=0 and time=9 minutes denoting the formation of hydroperoxides during photopolymerization of EDMA in the absence of PI under air. S10
Figure S15. Irgacure 819, its homolytic cleavage under light S10
Figure S16. Methacrylate double bond conversion for Eugenol-derived monomer versus irradiation time with Irgacure 819 and filter S11
Figure S17. Allylic and Propenyl double bond conversion for eugenol-derived monomers versus irradiation time with Irgacure 819 and filter S11

1. IR spectra of eugenol-derived methacrylates

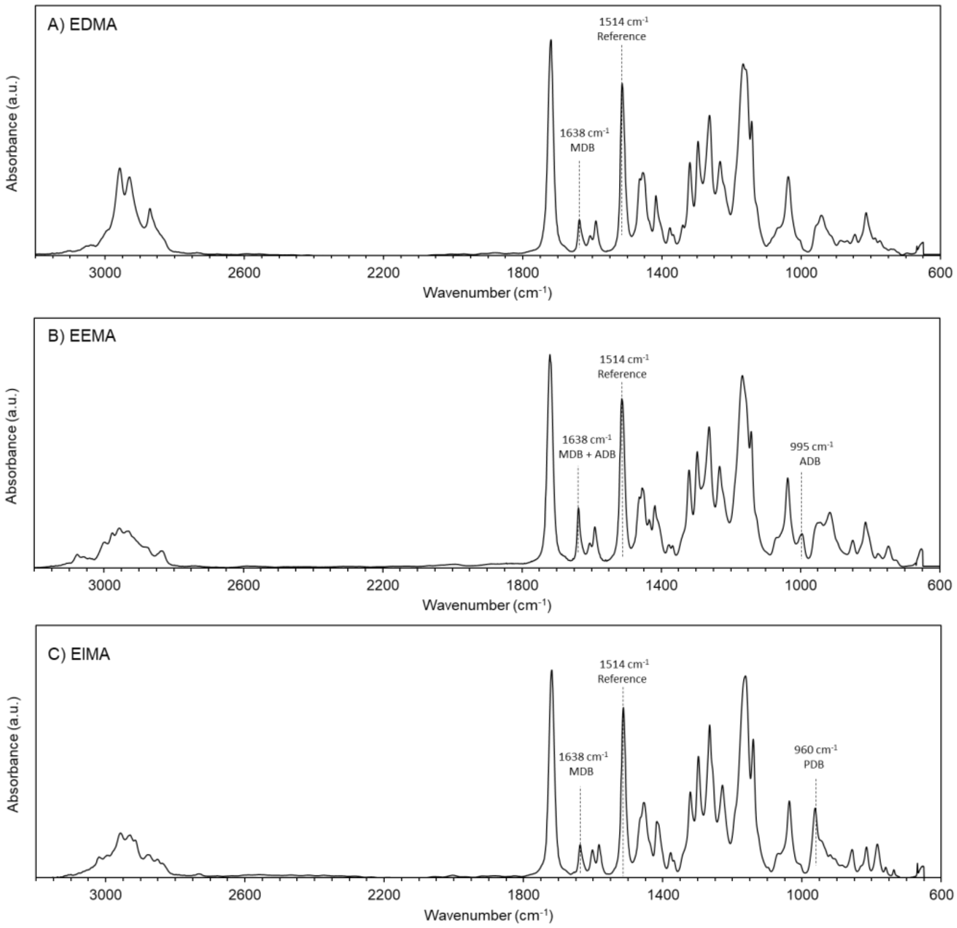

Figure S1. IR spectra in transmission of the different eugenol derived methacrylates: A) EDMA, B) EEMA and C) EIMA

2. Calculation of the conversion of the eugenol-derived monomers during photoinduced polymerization

As reported in the experimental part, IR spectra of the monomers were collected in real time during irradiation and the conversion of the double bonds was estimated according to Eq. S1:

$$Conversion\%_{t=x} = 100 \times \left(1 - \frac{\frac{A_{t=x}}{Ref.A_{t=x}}}{\frac{A_{t=0}}{Ref.A_{t=0}}}\right) \qquad \text{Eq. S1}$$

where A is the absorbance of the IR band of the functional group monitored during irradiation; Ref A is the absorbance of the band of the aromatic ring (C-C stretching) taken as a reference (1540 cm^{-1} to 1490 cm^{-1}).

For EDMA, the functional group monitored was the methacrylic double bond (MDB) band at 1638 cm^{-1}. For EIMA, both the methacrylic double bond (MDB) band at 1638 cm^{-1} and the propenyl double bond (PDB) band at 960 cm^{-1} were monitored.

In the case of EEMA, the band at *circa* 1638 cm^{-1} is not only due to the methacrylic double bond (MDB), but also to the allyl double bond (ADB). In fact, this ADB band is already present in the IR spectra of both eugenol and ethoxy eugenol (EE) (**Figure S2** and **Figure S3**), precursors of the methacrylated EEMA molecule.

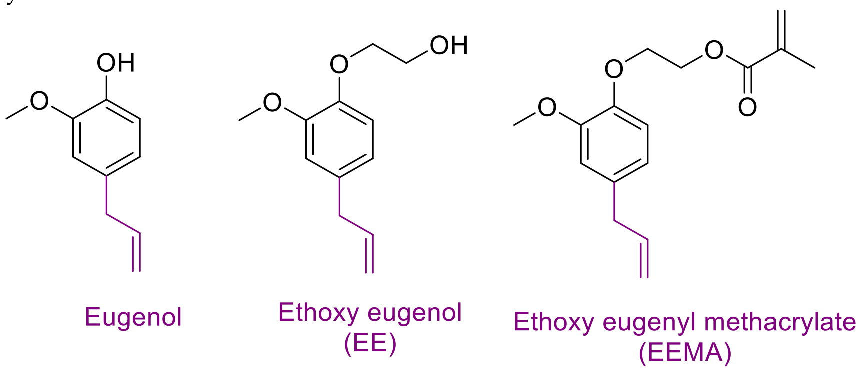

Figure S2. Eugenol, ethoxy eugenol and ethoxy eugenyl methacrylate

Transmission spectra of the precursos and monomers were done on a Thermo Scientific Nicolet 6700 FTIR apparatus in the 525–4000 cm^{-1} range, with 32 scan and a resolution of 2 cm^{-1}

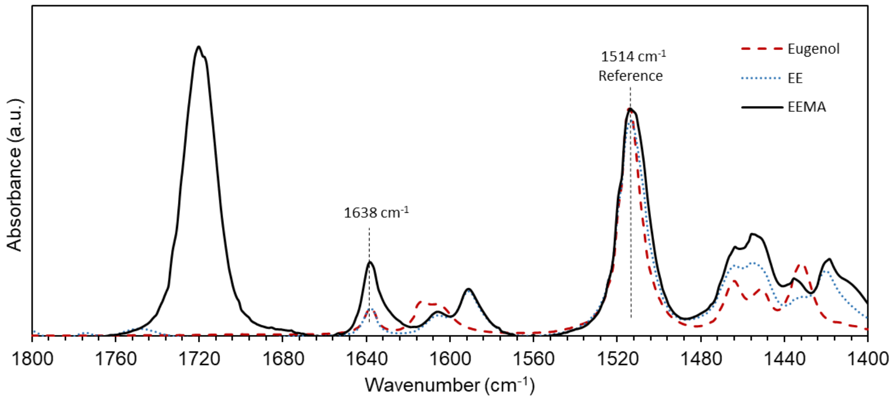

Figure S3. Transmission spectra of eugenol, ethoxyeugenol and ethoxy eugenyl methacrylate.

The overlap of the signals coming from methacrylic (MDB) and allylic (ADB) groups of EEMA clearly hinders the direct monitoring of the methacrylic double bond conversion through the area of the peak at 1638 cm^{-1} as conducted for EDMA and EIMA.

As the peaks from methacrylic and allylic groups are centered at the same wavenumber, thus superimposed, the deconvolution process of the band was not able to accurately separate the two contributions of MDB and ADB for EEMA at 1638 cm^{-1}. Therefore, another strategy was chosen. Spectra from EEMA precursors, i.e. eugenol and ethoxy eugenol EE (Figure S3) were recorded and processed as follows. The areas of the allyl group in eugenol and ethoxy eugenol were measured and normalized (using the reference peak at 1514 cm^{-1}) (Table S1). The normalized values 0.057 and 0.065 were averaged. The average value 0.061, was considered as representing the allylic contribution (ADB) to the band at 1638 cm^{-1} in the EEMA spectrum with respect to the area of the reference band (Eq. S2).

$$A_{ADB\ (1638cm^{-1})_{t=0}} = A_{Ref\ (1514cm^{-1})_{t=0}} \times 0.061 \qquad \text{Eq. S2}$$

And

$$\begin{aligned} A_{MDB\ (1638cm^{-1})_{t=0}} &= A_{peak\ (1638cm^{-1})_{t=0}} - A_{ADB\ (1638cm^{-1})_{t=0}} \\ &= A_{peak\ (1638cm^{-1})_{t=0}} - A_{Ref\ (1514cm^{-1})_{t=0}} \times 0.061 \end{aligned} \qquad \text{Eq. S2b}$$

Table S1. Normalization of the allylic band area for Eugenol and EE.

Band	Eugenol	EE
Area (a.u.): Peak 1638 cm^{-1}	1.042 (allylic)	0.558 (allylic)
Area (a.u.): Reference 1514 cm^{-1}	18.373 (aromatic)	8.540 (aromatic)
Normalized Area (a.u.) ($A_{ADB\ (1638cm^{-1})}$ / $A_{Ref\ (1514cm^{-1})}$)	0.057	0.065

The conversion of the methacrylic double bond (MDB) of EEMA was calculated as follows:

- The allylic group contribution (ADB) at any time in the composed peak at 1638 cm^{-1} is calculated using Eq. S3, used in the form of Eq. S4.
- The area corresponding to the allylic group contribution at the 1638 cm^{-1} peak at any given time, calculated by Eq. S4, is subtracted from the total area of the 1638 cm^{-1} peak to give the area corresponding to the MDB as shown in Eq. S5.
- Conversion of MDB at 1638 cm^{-1} is then calculated using reference peak as shown in Eq. S6.

$$R = \frac{A_{ADB\ (1638cm^{-1})_{t=0}}}{A_{ADB\ (995cm^{-1})_{t=0}}} = \frac{A_{Ref\ (1514cm^{-1})_{t=0}} \times 0.061}{A_{ADB\ (995cm^{-1})_{t=0}}} = \frac{A_{ADB\ (1638cm^{-1})_{t=x}}}{A_{ADB\ (995cm^{-1})_{t=x}}} \qquad \text{Eq. S3}$$

$$A_{ADB\ (1638cm^{-1})_{t=x}} = \frac{A_{Ref\ (1514cm^{-1})_{t=0}} \times 0.061}{A_{ADB\ (995cm^{-1})_{t=0}}} \times A_{ADB\ (995cm^{-1})_{t=x}} \qquad \text{Eq. S4}$$

$$\begin{aligned} A_{MDB\ (1638cm^{-1})_{t=x}} &= A_{peak\ (1638cm^{-1})_{t=x}} - A_{ADB\ (1638cm^{-1})_{t=x}} \\ &= A_{peak\ (1638cm^{-1})_{t=x}} - \frac{A_{Ref\ (1514cm^{-1})_{t=0}} \times 0.061}{A_{ADB\ (995cm^{-1})_{t=0}}} \\ &\quad \times A_{ADB\ (995cm^{-1})_{t=x}} \end{aligned} \qquad \text{Eq. S5}$$

$$EEMA\ MDB\ Conversion\ \%_{t=x} = 100 \times \left(1 - \frac{\frac{A_{MDB\ (1638cm^{-1})_{t=x}}}{A_{Ref\ (1514cm^{-1})_{t=x}}}}{\frac{A_{MDB\ (1638cm^{-1})_{t=0}}}{A_{Ref\ (1514cm^{-1})_{t=0}}}} \right) \qquad \text{Eq. S6}$$

$$= 100 \times \left(1 - \frac{\frac{A_{peak\ (1638cm^{-1})_{t=x}} - A_{ADB\ (1638cm^{-1})_{t=x}}}{A_{Ref\ (1514cm^{-1})_{t=x}}}}{\frac{A_{MDB\ (1638cm^{-1})_{t=0}}}{A_{Ref\ (1514cm^{-1})_{t=0}}}} \right)$$

$$= 100 \times \left(1 - \frac{\dfrac{A_{peak\,(1638cm^{-1})_{t=x}} - \dfrac{A_{Ref\,(1514cm^{-1})_{t=0}} \times 0.061}{A_{ADB\,(995cm^{-1})_{t=0}}} \times A_{ADB\,(995cm^{-1})_{t=x}}}{A_{Ref\,(1514cm^{-1})_{t=x}}}}{\dfrac{A_{MDB\,(1638cm^{-1})_{t=0}}}{A_{Ref\,(1514cm^{-1})_{t=0}}}} \right)$$

$$= 100 \times \left(1 - \frac{\dfrac{A_{peak\,(1638cm^{-1})_{t=x}} - \dfrac{A_{Ref\,(1514cm^{-1})_{t=0}} \times 0.061}{A_{ADB\,(995cm^{-1})_{t=0}}} \times A_{ADB\,(995cm^{-1})_{t=x}}}{A_{Ref\,(1514cm^{-1})_{t=x}}}}{\dfrac{A_{peak\,(1638cm^{-1})_{t=0}} - A_{Ref\,(1514cm^{-1})_{t=0}} \times 0.061}{A_{Ref\,(1514cm^{-1})_{t=0}}}} \right)$$

3. UV Spectra

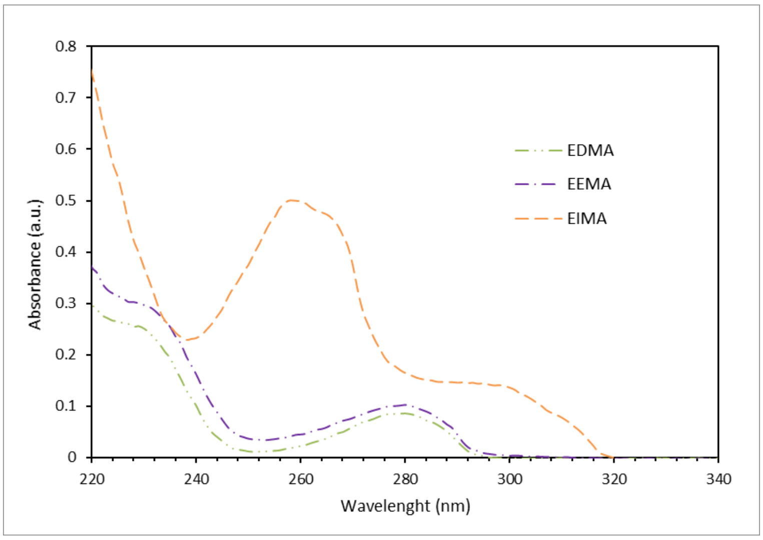

Figure S4. UV absorption spectra of the eugenol-derived methacrylates, in acetonitrile 0.002 wt%.

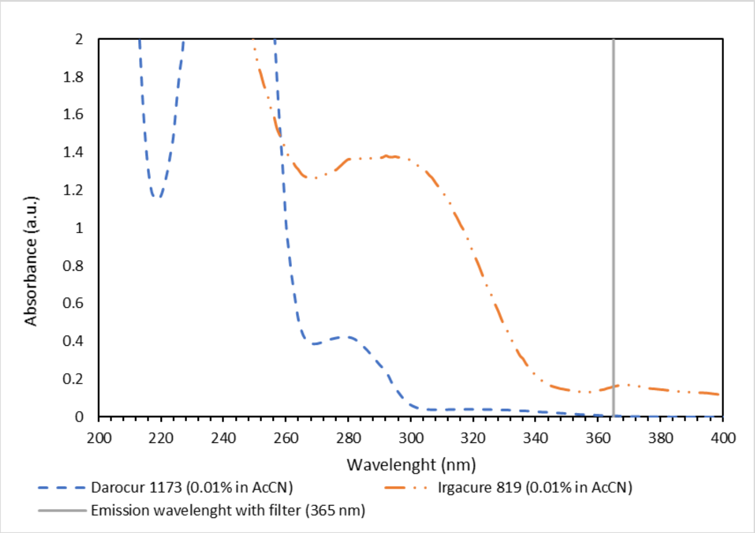

Figure S5. UV Absorption of photoinitiators.

4. DSC Measurements

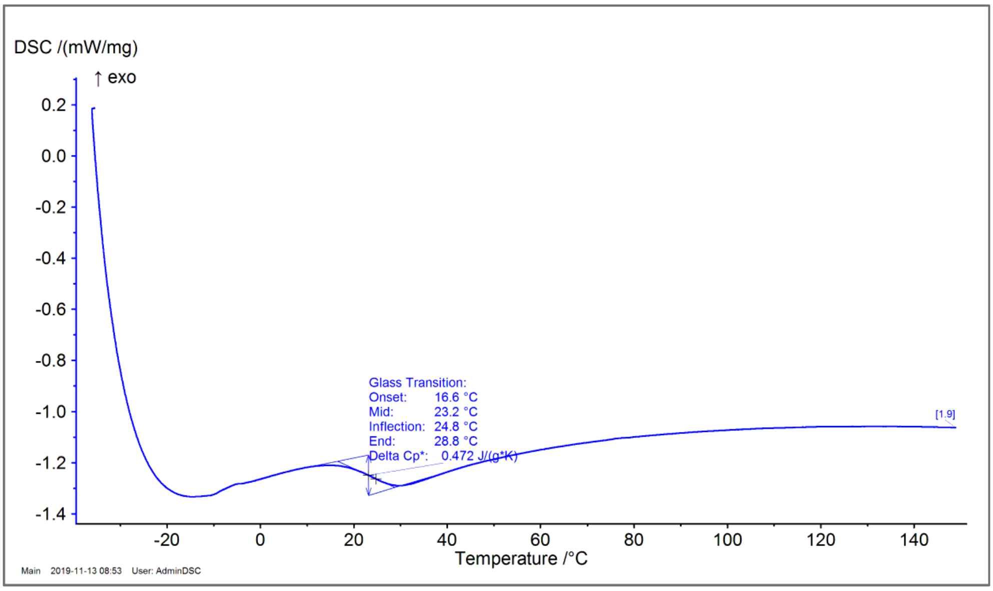

Figure S6. DSC measurement of poly(EDMA) obtained from photoinduced polymerization with Darocur 1173 (2% wbm) under nitrogen.

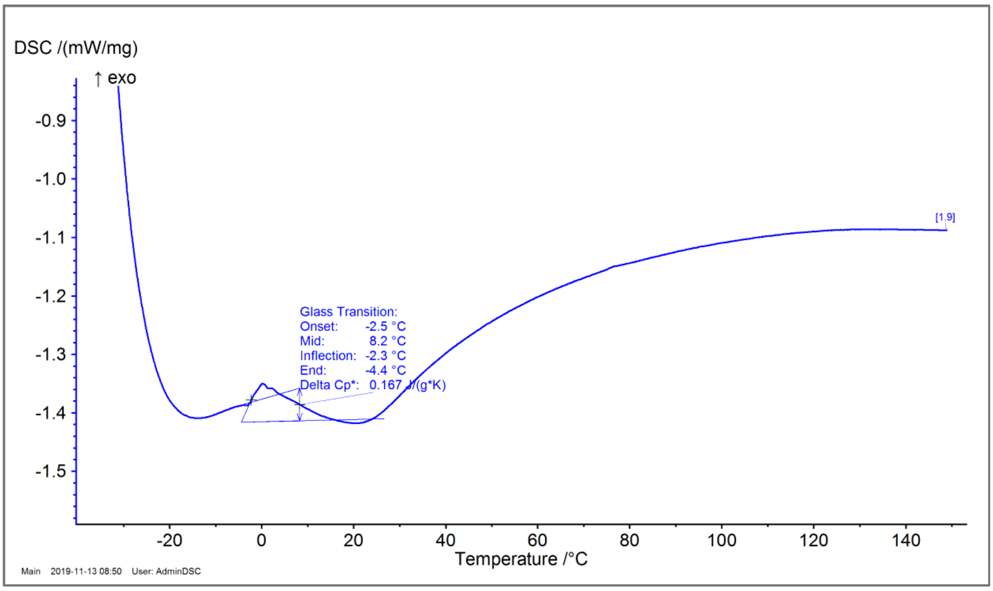

Figure S7. DSC measurement of poly(EDMA) obtained from photoinduced polymerization with Darocur 1173 (2% wbm) under air.

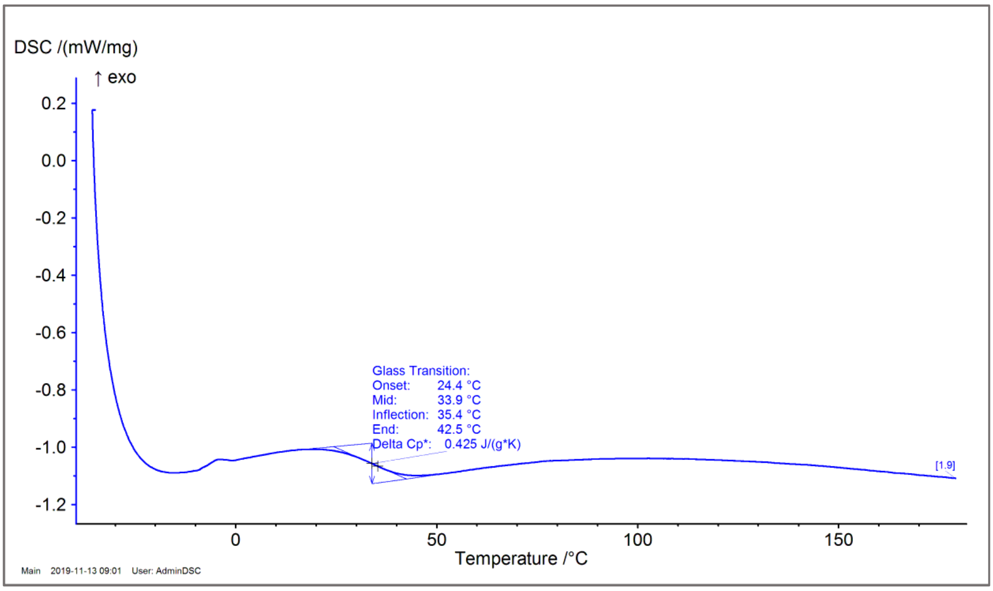

Figure S8. DSC measurement of poly(EEMA) obtained from photoinduced polymerization with Darocur 1173 (2% wbm) under nitrogen.

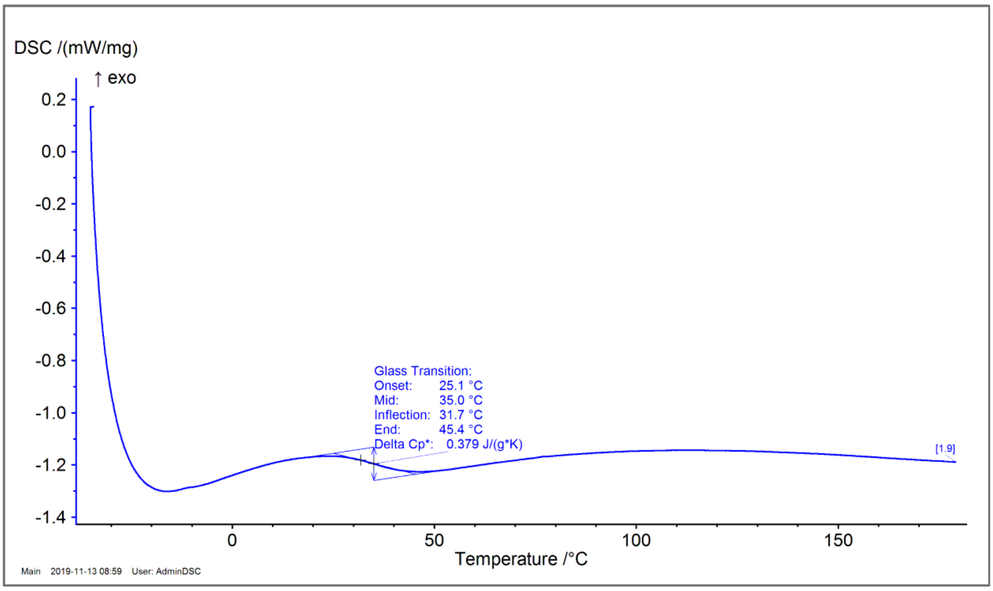

Figure S9. DSC measurement of poly(EEMA) obtained from photoinduced polymerization with Darocur 1173 (2% wbm) under air.

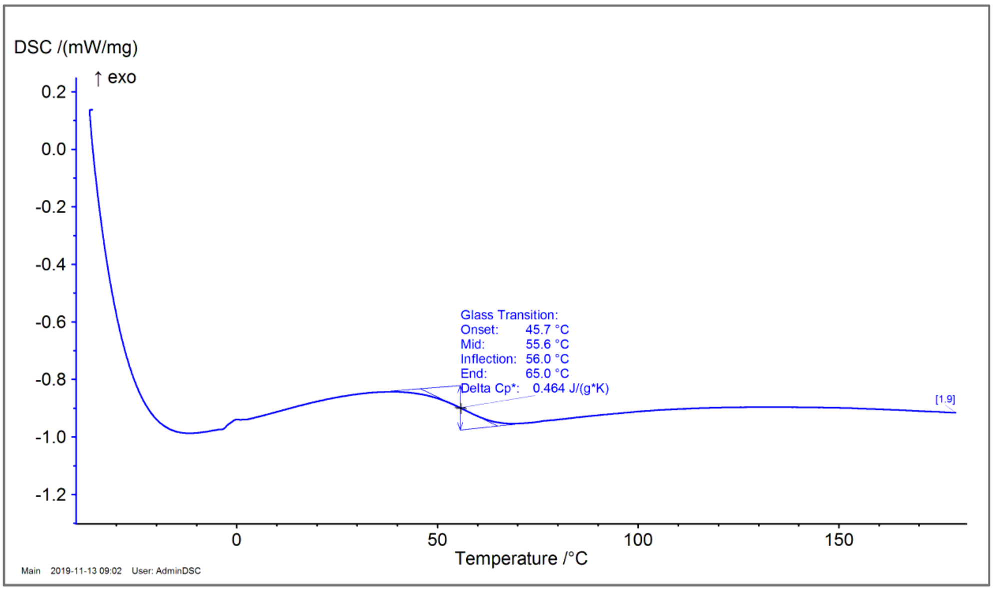

Figure S10. DSC measurement of poly(EIMA) obtained from photoinduced polymerization with Darocur 1173 (2% wbm) under nitrogen.

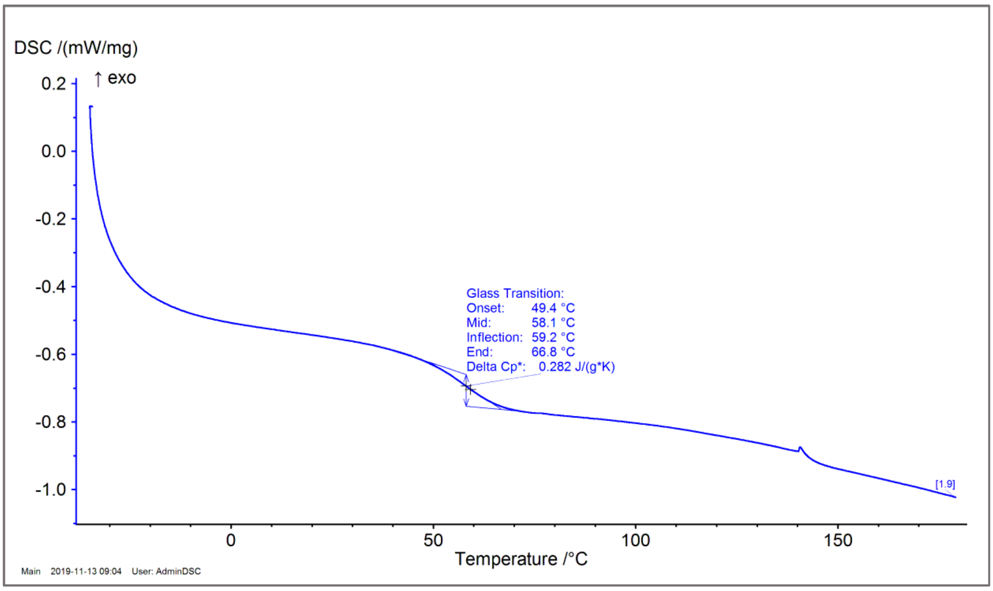

Figure S11. DSC measurement of poly(EIMA) obtained from photoinduced polymerization with Darocur 1173 (2% wbm) under air.

5. Hydroperoxide formation

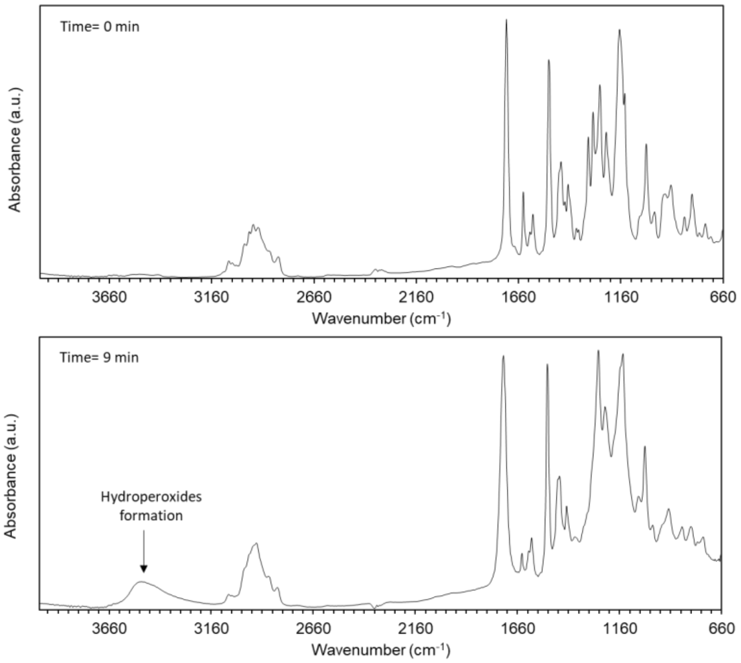

Figure S12. FT-IR spectra at time=0 and time=9 minutes denoting the formation of hydroperoxides during photopolymerization of EEMA in the absence of PI under air.

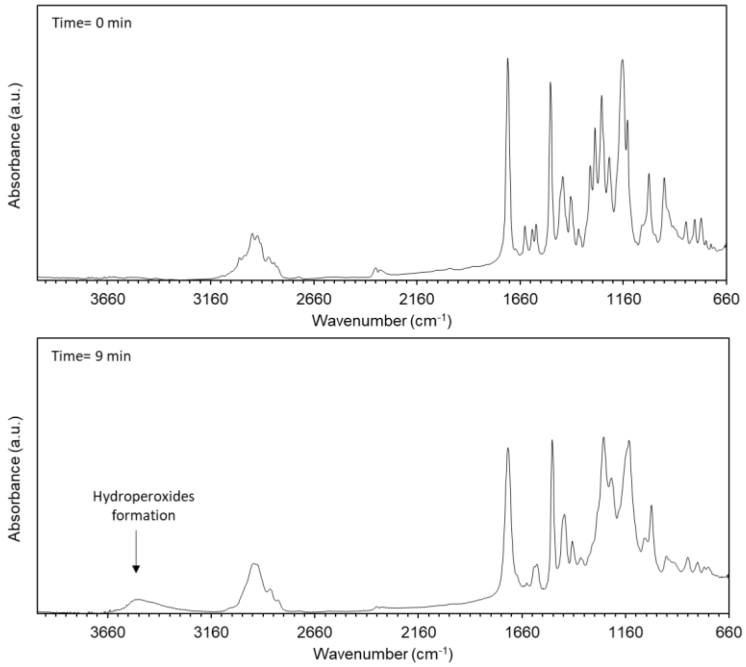

Figure S13. FT-IR spectra at time=0 and time=9 minutes denoting the formation of hydroperoxides during photopolymerization of EIMA in the absence of PI under air.

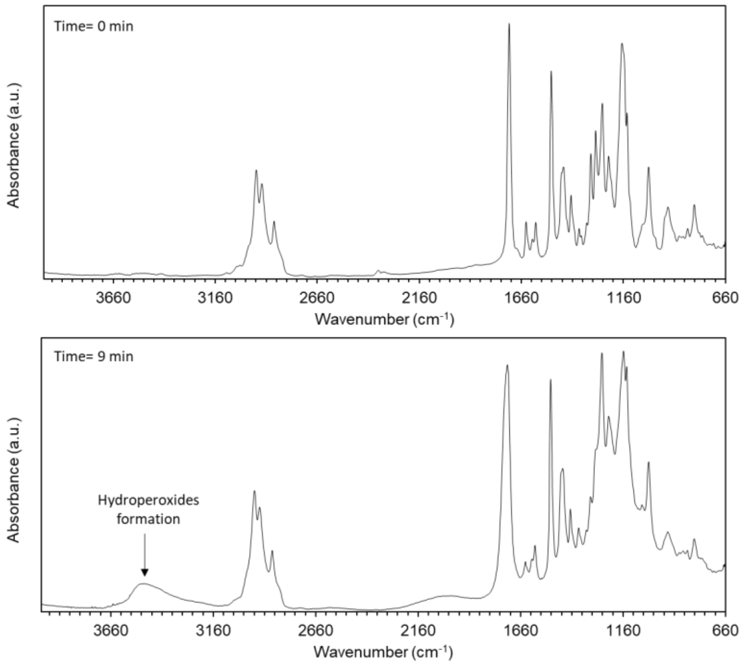

Figure S14. FT-IR spectra at time=0 and time=9 minutes denoting the formation of hydroperoxides during photopolymerization of EDMA in the absence of PI under air.

6. Photoinduced polymerization of eugenol-derived methacrylates with Irgacure 819.

Photoinduced polymerization of eugenol-derived monomers was done with Irgacure 819®. The homolytic cleavage of Irgacure 819® is shown in Figure S15 and its UV spectrum in Figure S4.

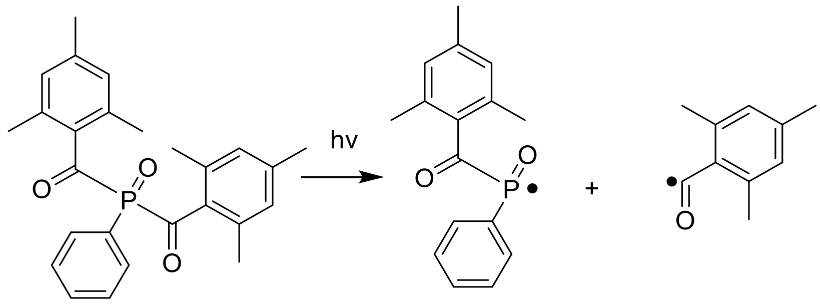

Figure S15. Irgacure 819, its homolytic cleavage under light

A behavior similar to that of Darocur 1173 was observed. Conversion of MDB, ADB and PDB for the respective monomers are presented in **Figure S16** and **Figure S17**. In the presence of oxygen, no significant reaction took place for EDMA and EEMA MDB (8% and 7% conversion respectively) while EIMA MDB reached a conversion of 40%. EIMA may be able to produce charge-transfer complexes leading to the formation of peroxides and radicals, allowing polymerization to proceed to a limited extent. In the absence of air, the conversion order was as follows: EDMA (96%) > EIMA (78%) >EEMA (76%). EDMA was the most reactive monomer (through the methacrylate double bond) as no secondary reactions are present. EEMA followed with only a slight reduction of polymerization rate due to the formation of highly stable bis-allylic radicals. Finally, EIMA came last due to cross-propagation between propenyl and methacrylate double bonds. This secondary reaction only marginally affected the methacrylate double bond conversion, which reached values very close to those of EEMA.

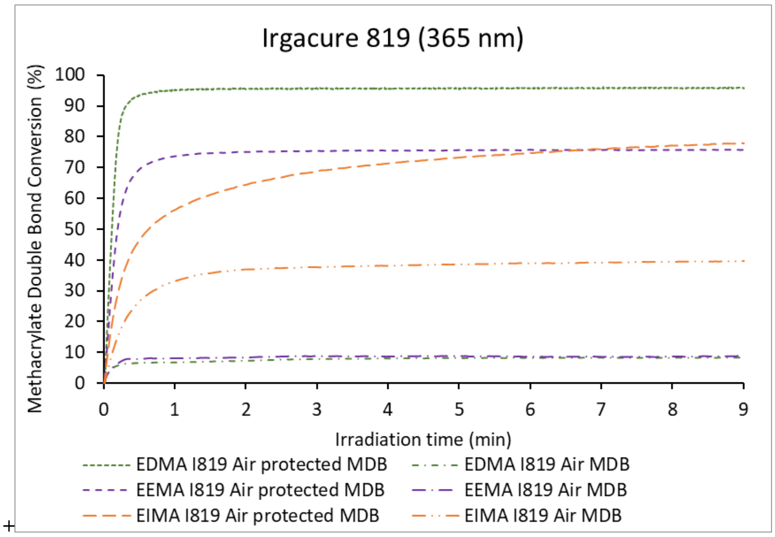

Figure S16. Methacrylate double bond conversion for Eugenol-derived monomer versus irradiation time with Irgacure 819 and filter

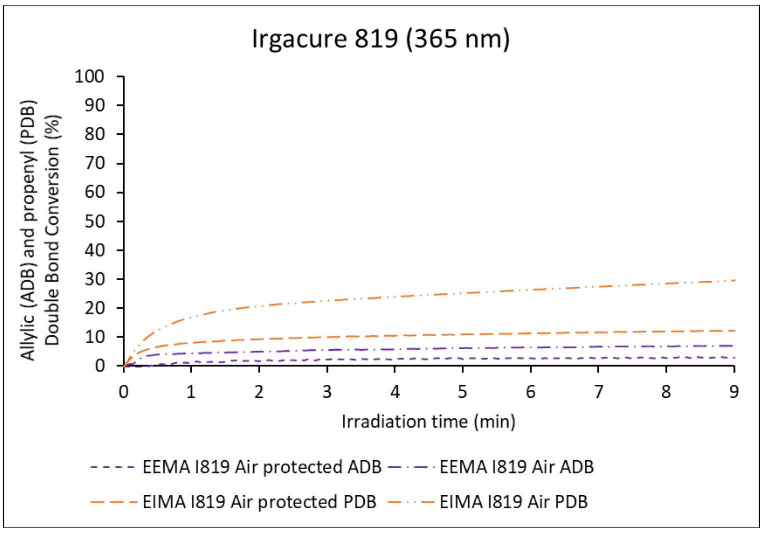

Figure S17. Allylic and Propenyl double bond conversion for eugenol-derived monomers versus irradiation time with Irgacure 819 and filter

7. Monomer conversion

Table S2. Monomer conversion of films used for polymer characterization (by ATR-FTIR).

Monomer	Condition	Darocur 1173	
		Upper side (contact with atmosphere) (%)	Lower side (contact with glass substrate) (%)
EDMA MDB	Air protected	100	100
EEMA MDB		85	85
EIMA MDB		99	98
EEMA ADB		27	0
EIMA PDB		96	99
EDMA MDB	Air	91	92
EEMA MDB		81	57
EIMA MDB		99	99
EEMA ADB		74	7
EIMA PDB		94º	65
